# Unbalanced Risk of Pulmonary Tuberculosis in China at the Subnational Scale: Spatiotemporal Analysis

**DOI:** 10.2196/36242

**Published:** 2022-07-01

**Authors:** Maogui Hu, Yuqing Feng, Tao Li, Yanlin Zhao, Jinfeng Wang, Chengdong Xu, Wei Chen

**Affiliations:** 1 State Key Laboratory of Resources and Environmental Information System Institute of Geographical Sciences and Natural Resources Research Beijing China; 2 College of Resources and Environment University of Chinese Academy of Sciences Beijing China; 3 National Center for Tuberculosis Control and Prevention Chinese Center for Disease Control and Prevention Beijing China

**Keywords:** pulmonary tuberculosis, infectious disease, pattern, notification rates, Bayesian, spatiotemporal pattern, tuberculosis, public health, China, disease burden, spatial data, regional inequality, risk, TB, unbalanced, notification data, trend, cases, incidence

## Abstract

**Background:**

China has one of the highest tuberculosis (TB) burdens in the world. However, the unbalanced spatial and temporal trends of TB risk at a fine level remain unclear.

**Objective:**

We aimed to investigate the unbalanced risks of pulmonary tuberculosis (PTB) at different levels and how they evolved from both temporal and spatial aspects using PTB notification data from 2851 counties over a decade in China.

**Methods:**

County-level notified PTB case data were collected from 2009 to 2018 in mainland China. A Bayesian hierarchical model was constructed to analyze the unbalanced spatiotemporal patterns of PTB notification rates during this period at subnational scales. The Gini coefficient was calculated to assess the inequality of the relative risk (RR) of PTB across counties.

**Results:**

From 2009 to 2018, the number of notified PTB cases in mainland China decreased from 946,086 to 747,700. The average number of PTB cases in counties was 301 (SD 26) and the overall average notification rate was 60 (SD 6) per 100,000 people. There were obvious regional differences in the RRs for PTB (Gini coefficient 0.32, 95% CI 0.31-0.33). Xinjiang had the highest PTB notification rate, with a multiyear average of 155/100,000 (RR 2.3, 95% CI 1.6-2.8; *P*<.001), followed by Guizhou (117/100,000; RR 1.8, 95% CI 1.3-1.9; *P*<.001) and Tibet (108/100,000; RR 1.7, 95% CI 1.3-2.1; *P*<.001). The RR for PTB showed a steady downward trend. Gansu (local trend [LT] 0.95, 95% CI 0.93-0.96; *P*<.001) and Shanxi (LT 0.94, 95% CI 0.92-0.96; *P*<.001) experienced the fastest declines. However, the RRs for PTB in the western region (such as counties in Xinjiang, Guizhou, and Tibet) were significantly higher than those in the eastern and central regions (*P*<.001), and the decline rate of the RR for PTB was lower than the overall level (*P*<.001).

**Conclusions:**

PTB risk showed significant regional inequality among counties in China, and western China presented a high plateau of disease burden. Improvements in economic and medical service levels are required to boost PTB case detection and eventually reduce PTB risk in the whole country.

## Introduction

Tuberculosis (TB), an infectious disease caused by the bacterium *Mycobacterium tuberculosis*, is one of the top 10 causes of death worldwide. There were approximately 10 million new TB cases worldwide in 2019 and 1.4 million people died due to TB [1]. In the 1990s, China implemented the directly observed treatment, short-course (DOTS) approach, as recommended by the World Health Organization. Additionally, a series of major measures have been taken, including the formulation and implementation of TB prevention and control plans and the implementation of international collaborative projects for TB control. China has achieved the United Nations’ 2015 Millennium Development Goals 5 years ahead of schedule and has made a significant contribution to global TB prevention and control [2-4]. However, there are still challenges in the prevention of TB in China. Specifically, in 2019, China had approximately 840,000 new TB cases, accounting for 8.4% of the global total and ranking third among the 30 countries with a high TB burden [1]. In terms of the 2030 sustainable development goals (SDGs), preventing and managing TB in China is a challenging mission.

Despite some studies on the TB risk in China at the national and provincial levels, there has been a lack of evaluation of the unbalanced risk of pulmonary tuberculosis (PTB) as well as the trend of the risk at fine scales from long-term series in China in recent years [5-12]. A county-level study of PTB risk in China can reveal variations in the severity and efficacy of PTB prevention and control in different regions (provinces, municipalities, and autonomous regions) on a fine scale.

Therefore, the aim of this study was to investigate the unbalanced risks of PTB at different levels and how they evolved in both temporal and spatial aspects using PTB notification data from 2851 counties over a decade in China. These findings could be beneficial to optimize the distribution of health resources in China for TB prevention and control.

## Methods

### Data Sources and Definition of PTB Notification Rate

TB is one of the 40 notifiable infectious diseases in China. To improve the timely diagnosis, treatment, and supervision of TB patients nationwide, the Chinese Ministry of Health launched the National Tuberculosis Information Management System (TBIMS) in 2005, which records detailed information on TB patients, including outpatient information, case information, treatment, and supervision, as well as information on TB planning and management. In China, all diagnosed TB (any kind) should be recorded to the TBIMS according to the national TB prevention and control guidelines; thus, all active TB is included in the system such as bacterially confirmed or clinically diagnosed TB and extrapulmonary TB. All TB prevention and control facilities across the country can use the system to record TB case information and planning activity data in real time. This can be used to increase the detection rate of TB and strengthen TB patient management, as well as to control and eventually eliminate TB.

China’s administrative regions are generally divided into the following four levels: provincial, prefectural, county, and township levels. Counties are the basis of local government in the administrative division of Chinese society. There are specialized institutions for TB prevention and control at the provincial, prefectural, and county levels in China, including TB clinics, disease prevention and control centers, and TB designated hospitals.

In this study, the numbers of notified PTB cases and the populations of the 2851 county-level areas in mainland China from 2009 to 2018 were collected from the TBIMS. The annual PTB notification rate in a region was defined as the ratio of the annual number of notified PTB cases and the total population at the end of the year in the region.

### Spatiotemporal Trend Modeling of the PTB Notification Rate

A Bayesian spatiotemporal model was built to analyze spatiotemporal patterns of PTB notification rates from 2009 to 2018. The spatiotemporal process of the PTB notification rate was decomposed into three components: the spatial random effect, overall time trend, and spatiotemporal interaction effect [13-15]. A Poisson regression model connected by a logarithmic function was used to model the process based on count data [16,17]. The Poisson log-normal model with the spatiotemporal effect is as follows:

*y_it_*=Poisson(*μ_it_*) **(1)**

*μ_it_*=*e_it_θ_it_*** (2)**

log(*θ_it_*)=*α*+*s_i_*+(b_0_t^*^+v*_t_*)+b_1i_t^*^+*ε_it_*
** (3)**

where *y_it_* denotes the number of notified PTB cases in year *t* in the *i*th county, *μ_it_* is the expected number notified PTB cases, *α* is the overall average level of the PTB notification rate in mainland China from 2009 to 2018, and *s_i_* is the relative risk (RR) for PTB in the *i*th county during the study period; exp(*s_i_*)>1 indicates that the RR for PTB in the *i*th county is higher than the overall average across mainland China, while exp(*s_i_*)<1 means that the RR for PTB in the *i*th county is lower than the overall average. The time span relative to the middle time point (*t_mid_*) of the study period is represented by t^*^=*t*–*t_mid_*. b_0_t^*^+*v_t_* describes the overall time trend of the RR, consisting of a linear trend b_0_t^*^ and a time-random effect *v_t_*, allowing the overall time trend to show a nonlinear change. The spatiotemporal interaction term b_1i_t^*^ represents the spatial variation in the time trend. *b_1i_* measures the local trend decomposed from the overall time trend. If exp(*b_1i_*)>1, the *i*th county has a stronger local trend (LT) than the global overall trend. Otherwise, exp(*b_1i_*)<1 indicates that the *i*th county has a weaker LT than the global overall trend. The *ε_ij_* item is an unstructured random effect in the model.

For each model parameter, prior distributions were allocated. A uniform distribution was assigned to the intercept term α. We selected the Besag-York-Mollie (BYM) model to reveal the overall spatial random effect *s_i_* [18,19]. The intrinsic conditional autoregressive (ICAR) prior with a spatial adjacency matrix *W* was adopted for the spatial structure, where *wij*=1 if the *i*th and *j*th counties are neighborhoods and *w_ij_*=0 otherwise. The ICAR prior implies that adjacent regions tend to have similar overall notification rates. The same BYM prior was used for the parameter *b*_1*i*_. The temporal random effect *v_t_* was assumed to follow a Gaussian distribution as *v_t_*~*N*(0, *σ*^2^*_v_*). Finally, *ε_ij_* followed the distribution *ε_ij_~N*(*σ*^2^*_ε_*). As suggested by Gelman [20], a strictly positive half Gaussian prior *N_+∞_*(0, 10) was assigned to parameters of random-effect standard deviations, including *σ_v_* and *σ_ε_*.

We implemented the model in WinBUGS, a software program specifically designed for Bayesian analysis [21]. Markov chain Monte Carlo (MCMC) simulations were used to obtain posterior distributions of model parameters. Specifically, with different starting values, we ran two MCMC chains for each model with 200,000 iterations. The first 150,000 iterations were burned in. Every 10th iteration for the remaining 50,000 MCMC iterations was retained to make inferences. The Gelman-Rubin statistic was used to test convergence [22]. The Gelman-Rubin statistic values for all parameters in this study were below 1.05, indicating that the model converged.

### Inequality Measurement: Gini Coefficient of PTB Risk

To assess the inequality of the RR of PTB across counties, we computed the Gini coefficient using the formula:

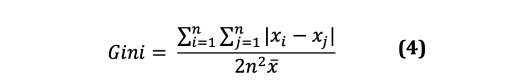



where *x* is the RR, and *i* and *j* indicate counties. As shown by equation (4), the Gini coefficient can be interpreted as the average relative differences between all pairs of counties.

## Results

### Temporal Trend and Spatial Distribution of the PTB Notification Rate

**Figure 1:**
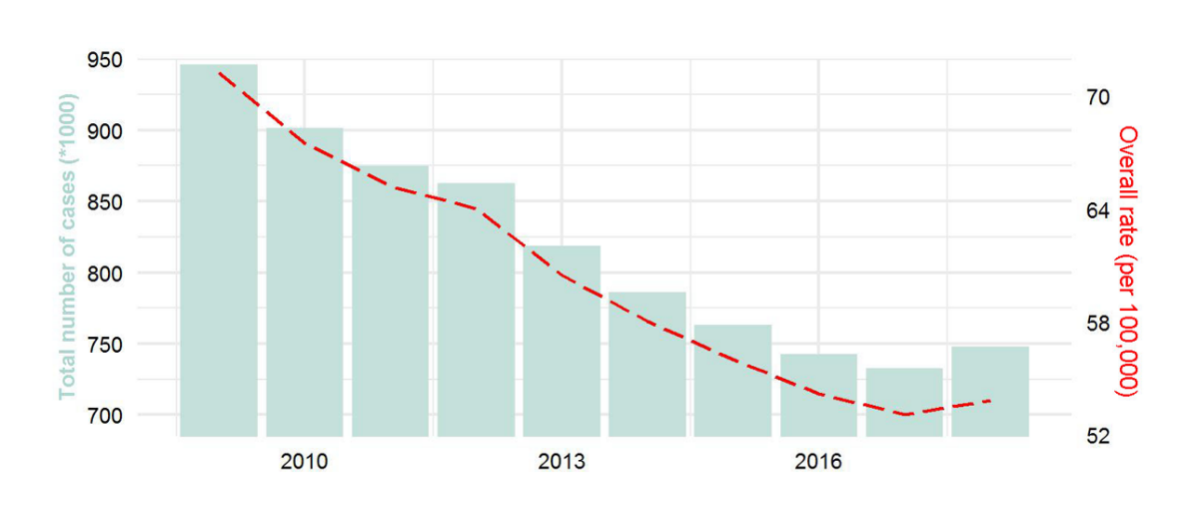
Temporal trend in the number of notified pulmonary tuberculosis cases and the overall rates in mainland China from 2009 to 2018.

**Figure 2:**
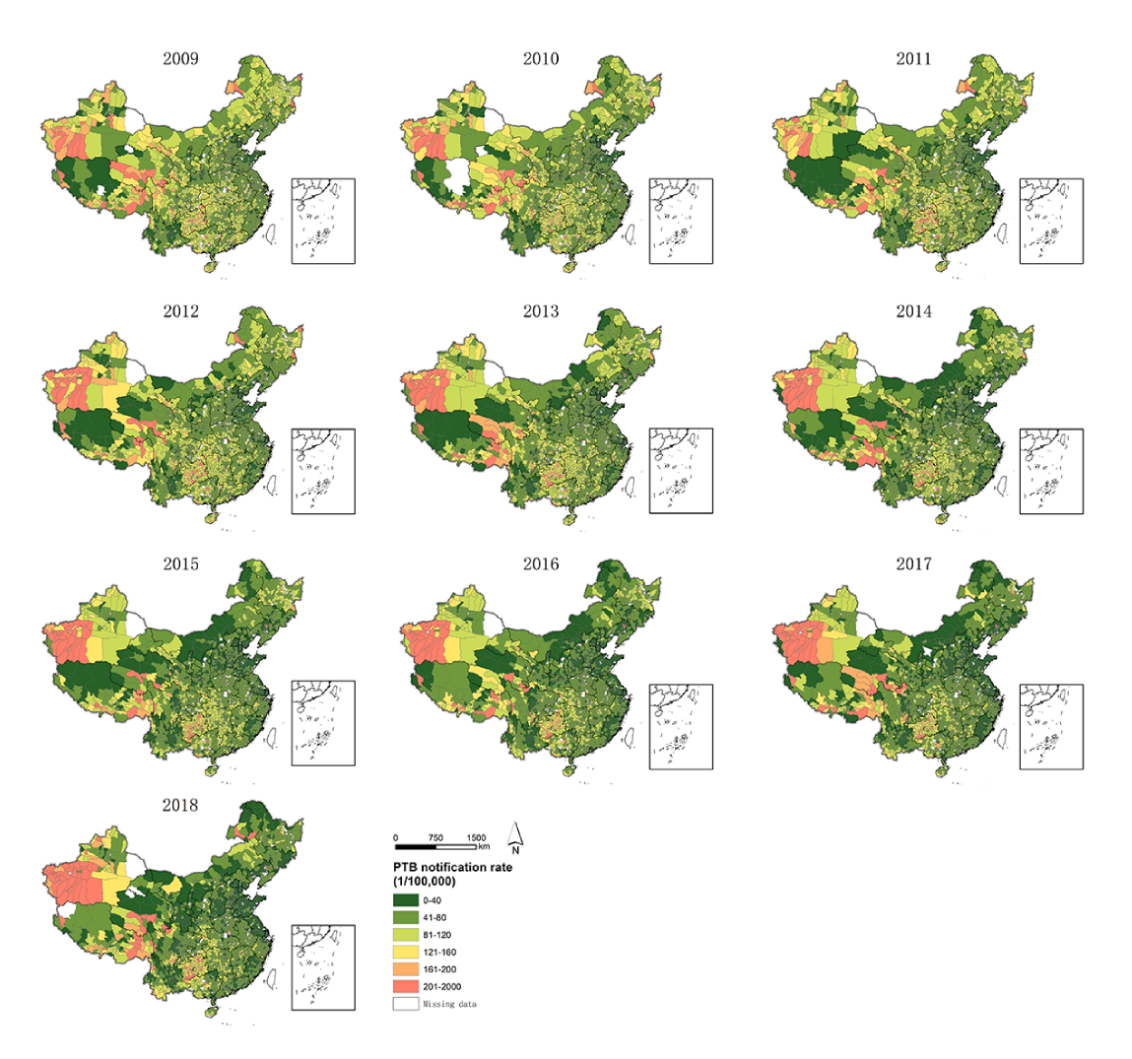
Spatial distributions of pulmonary tuberculosis (PTB) notification rates in counties of 31 provinces in mainland China from 2009 to 2018.

The number of notified PTB cases in mainland China decreased from 946,086 to 747,700 between 2009 and 2018, showing a downward trend (Table 1, Figure 1). In 2017, the number of notified PTB cases was at its lowest (732,612). The average number of PTB cases in counties decreased from 346.2 to 276.8, with the lowest reported in 2017 (270.5). The average number of PTB cases in counties was 301 (SD 26). The overall average notification rate in mainland China was 60 (SD 6) per 100,000 people. The rates of PTB notifications in China’s provinces varied significantly (Figure 2), with the rate in the western region being higher than those in the eastern and central regions. Western provinces, including Xinjiang, Tibet, Guizhou, and Guangxi, had multiyear overall notification rates higher than 90/100,000. Xinjiang had the highest PTB notification rate, with a multiyear average of 155/100,000, followed by Guizhou (117/100,000) and Tibet (108/100,000). The lowest PTB notification rates were reported in Beijing and Tianjin, with multiyear overall notification rates of 23 and 22 per 100,000, respectively. During the study period, notification rates declined in most provinces, but increased in some western provinces such as Xinjiang, Yunnan, and Qinghai. The rates in Gansu and Shanxi decreased by more than 50%, while the rates in Yunnan and Qinghai increased by 10%-15%.

**Table 1:** Summary statistics of the number of pulmonary tuberculosis (PTB) cases in counties.

Year	Total PTB cases (N=8,176,058), n (%)	Mean (SD)	Range				
			Minimum	P25^a^	P50^b^	P75^c^	Maximum
2009	946,086 (11.57)	346.2 (276.95)	0	149	275	466	2888
2010	901,189 (11.02)	330.3 (274.56)	0	144	258	439.2	3849
2011	874,759 (10.70)	321.6 (273.46)	1	139	251.5	432	4364
2012	862,579 (10.70)	316.8 (278.50)	1	131	246	424	4125
2013	818,876 (10.02)	301.7 (267.18)	1	124	232	410	3656
2014	786,256 (9.62)	289.9 (261.73)	1	113	220	389	3704
2015	763,230 (9.33)	282.3 (263.03)	2	108.8	209.5	378.2	3351
2016	742,771 (9.08)	274.5 (259.55)	1	106	207	368.5	3274
2017	732,612 (8.96)	270.5 (263.80)	1	104	204.5	358	4007
2018	747,700 (9.14)	276.8 (343.34)	0	98	204	349	7797

^a^P25: 25th percentile.

^b^P50: 50th percentile.

^c^P75: 75th percentile.

The spatial distributions of PTB notification rates within each province were also unbalanced with significant heterogeneity. The notification rates in southwestern Xinjiang, Guizhou, and southeastern Tibet were generally high in most years. The notification rate in southern Xinjiang increased dramatically. In contrast, the rates in counties in Gansu, northeastern China, central Inner Mongolia, eastern Guangxi, Shandong, Jiangsu, and Sichuan decreased significantly (Figure 2).

### Spatial Relative Risk and Temporal Trend of PTB

**Figure 3:**
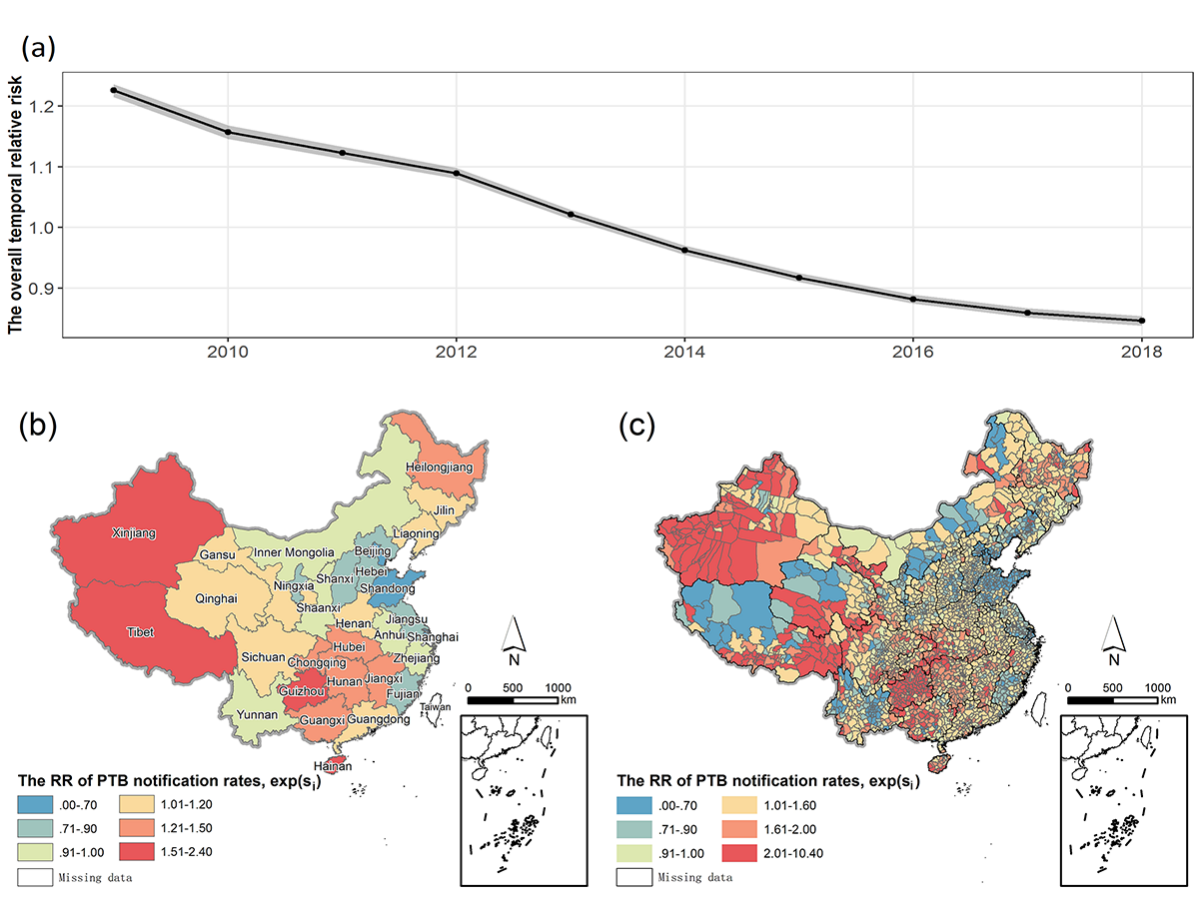
The relative risk (RR) for pulmonary tuberculosis (PTB) in mainland China from 2009 to 2018. (a) Overall temporal trend (95% CI); (b) RR at the province level; (c) RR at the county level. s_i_ is the RR for PTB in the ith county during the study period.

The RR for PTB showed a steady downward trend (Figure 3a), but the rate of decline gradually slowed in later years. There were obvious regional differences in the RRs for PTB, with those in the western region being significantly higher than those in the eastern and central regions (*P*<.001). Provinces were divided into three categories according to the RR values, represented by the item exp(*s_i_*) in the model (Figure 3b). The red and orange provinces (RR≥1.2) in Figure 3b had significantly higher RRs than the overall average. Xinjiang (RR 2.3, 95% CI 1.6-2.8; *P*<.001), Tibet (RR 1.7, 95% CI 1.3-2.1; *P*<.001), Guizhou (RR 1.8, 95% CI 1.3-1.9; *P*<.001), and Hainan (RR 1.7, 95% CI 1.3-2.1; *P*=.003) had the highest RRs, followed by Hubei, Chongqing, Hunan, Jiangxi, Guangxi, and the three northeastern provinces. The provinces indicated in blue and light blue (RR≤0.9) in Figure 3b had significantly lower RRs than the overall average. Beijing (RR 0.4, 95% CI 0.3-0.5; *P*<.001), Tianjin, Shanghai, and Shandong had the lowest RRs, followed by Hebei, Shanxi, Ningxia, Jiangsu, and Fujian. The RRs for PTB in other regions were similar to the overall average of mainland China (0.9<RR<1.2).

RRs were also spatially distributed heterogeneously at the county level within provinces (Figure 3c). In Xinjiang, 18% of counties had RRs<1, and they were located in the north-central region; 55% of counties had RRs>2, and most of them clustered in the south and north regions. The RRs for PTB in Tibet increased from northwest to southeast. Counties with RRs>2 (accounting for 45% of the total counties in Tibet) were distributed in the southeast. The classification based on the posterior distribution is shown in Multimedia Appendix 1.

### Local Trends in the RR of PTB

**Figure 4:**
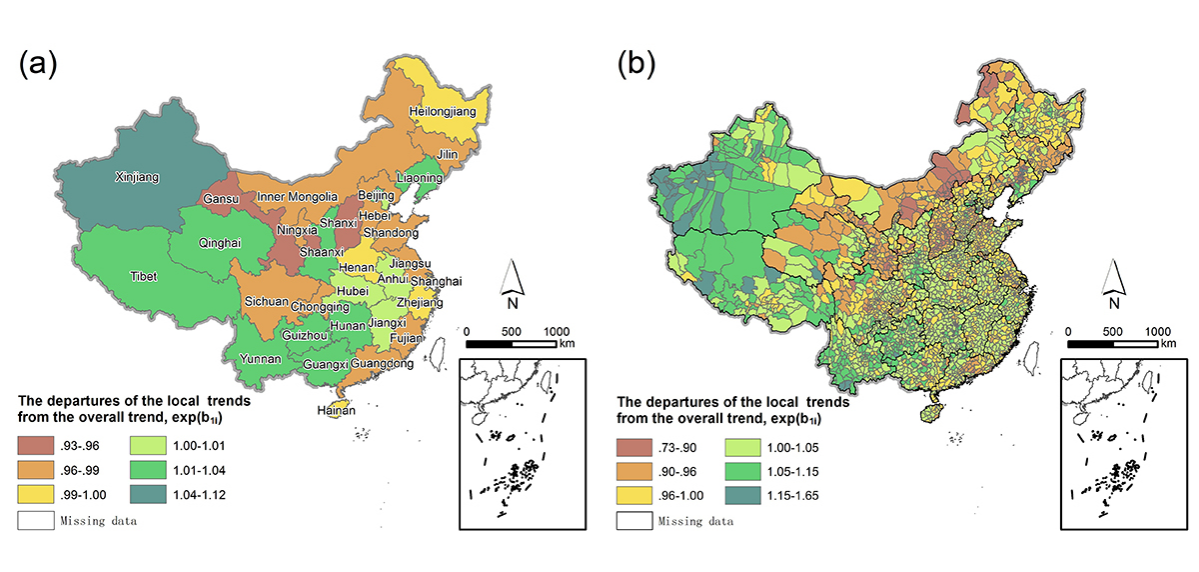
Distribution of the departures of the local trends from the overall trend in mainland China from 2009 to 2018. (a) The departures of the local trends from the overall trend at the province level. (b) The departures of the local trends from the overall trend at the county level.

In different regions of China, the decline rate of the RR varied significantly (Figure 4). The decline in the RR in the western region of China was generally lower than those in the central and eastern regions (*P*<.001). In mainland China, Xinjiang experienced the slowest RR decline, followed by Qinghai, Guizhou, Yunnan, Liaoning, and Beijing, all of which were slower than the overall trend. Gansu (LT 0.95, 95% CI 0.93-0.96; *P*<.001) and Shanxi (LT 0.94, 95% CI 0.92-0.96; *P*<.001) experienced the fastest declines, followed by Inner Mongolia (LT 0.97, CI 0.95-0.99, *P*<.001), Jilin (LT 0.96, 95% CI 0.95-0.98; *P*<.001), and Jiangsu (LT 0.97, 95% CI 0.95-0.99; *P*<.001), all of which were faster than the overall trend. At the county level, the proportion of counties with exp(*b*_1*i*_)>1 in Xinjiang, Guizhou, and Yunnan provinces was greater than 90%, and the proportion of counties with exp(*b*_1*i*_)<1 in Gansu and Shanxi provinces was 95%, displaying a fast decline.

**Figure 5:**
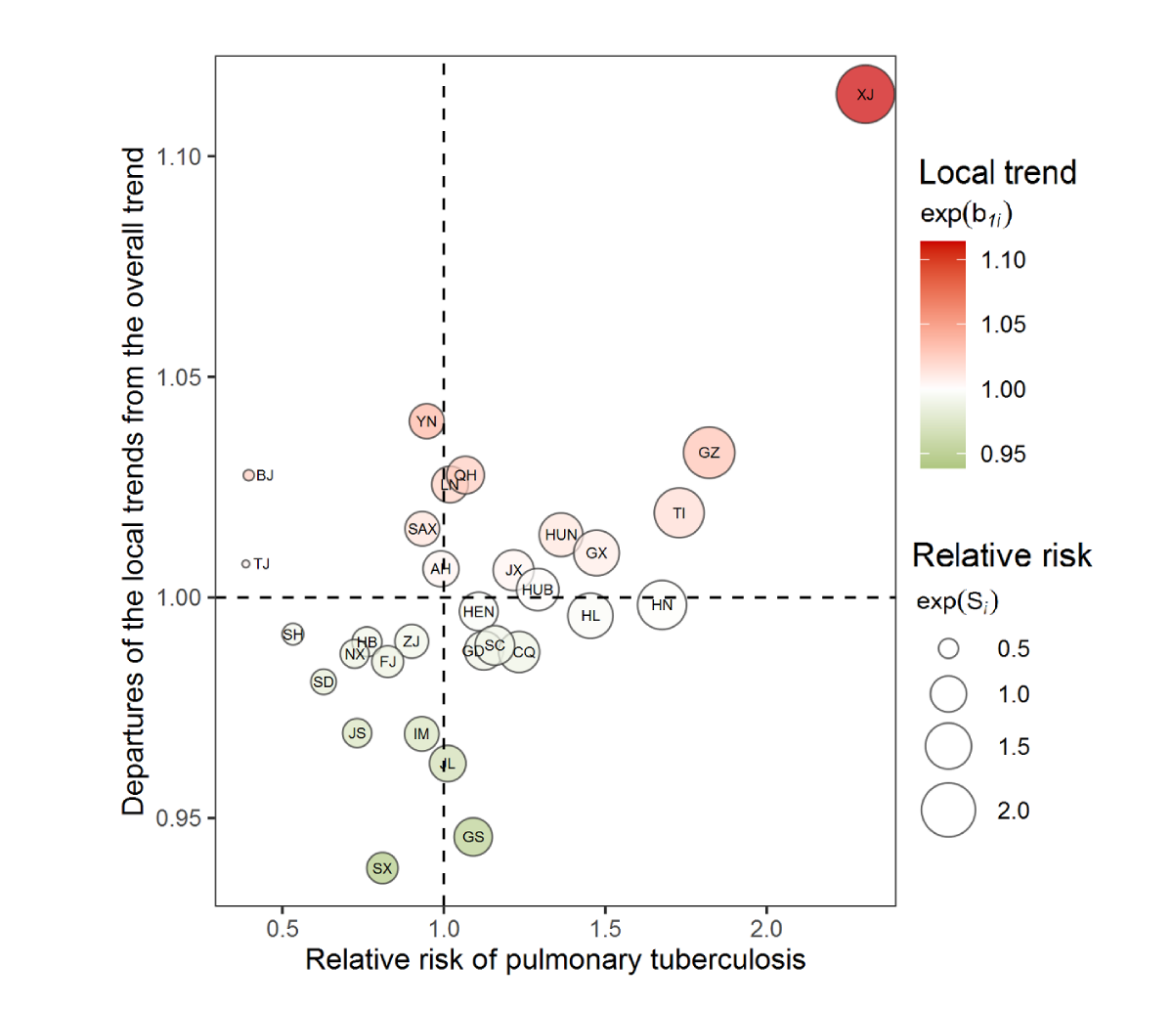
The severity and efficacy of pulmonary tuberculosis prevention and control in different provinces. Upper left quadrant: Anhui (AH), Beijing (BJ), Shaanxi (SAX), Tianjin (TJ), Yunnan (YN). Upper right quadrant: Guangxi (GX), Guizhou (GZ), Hubei (HUB), Hunan (HUN), Jiangxi (JX), Liaoning (LN), Qinghai (QH), Tibet (TI), Xinjiang (XJ). Lower left quadrant: Fujian (FJ), Hebei (HB), Inner Mongolia (IM), Jiangsu (JS), Ningxia (NX), Shandong (SD), Shanghai (SH), Shanxi (SX), Zhejiang (ZJ). Lower right quadrant: Chongqing (CQ), Gansu (GS), Guangdong (GD), Hainan (HN), Heilongjiang (HL), Henan (HEN), Jilin (JL), Sichuan (SC).

A summary of the provincial overall RRs of PTB and their departures from the country overall RR is shown in Figure 5 to compare the relative severity of PTB in different provinces. The bottom left quadrant includes provinces with lower RR and a faster decline in RR for PTB than the overall trend, such as Shanxi, Jiangsu, and Shandong. In contrast, the top right quadrant clusters provinces that had higher RRs and a slower decline in RR for PTB than the overall trend, such as Xinjiang, Guizhou, and Tibet.

## Discussion

We used the number of PTB cases at the county level from 2009 to 2018 to analyze the risk of PTB over a decade in China. The overall average notification rate was 60 (SD 6) per 100,000 people. The RRs for PTB showed a steady downward trend. China, which has one of the world’s highest TB burdens, still faces many TB-related public health challenges. Although the TB epidemic in China has decreased significantly in recent years, the imbalance in the burden of TB among different regions is severe, according to the fifth national TB prevalence sampling survey [2]. Generally, the TB prevalence in western regions is higher than those in eastern and central regions, and the TB prevalence is higher in rural areas than in urban areas. Discovering the unevenness of PTB risk between regions and the variability of its trends is important for allocating medical resources for PTB and assessing the efficiency of treatment.

China issued the “Stop Tuberculosis Action Plan (2019-2022)” to further promote the end of the TB epidemic. The plan clarifies the goals and directions of China’s recent TB control program [23]. The notification rate of PTB in mainland China showed a downward trend from 2009 to 2018. This phenomenon indicated that the comprehensive implementation of the national TB prevention and control plan had a significant effect, although the declining PTB trend has gradually slowed in recent years.

The spatial distribution of TB is related to the geographical location, economy, population, and environment, and its transmission is characterized by a spatial clustering distribution [5,10,11,24]. The results showed that the RR for PTB in the western region of China was significantly higher than those in the eastern and central regions. The reason might be that patients with PTB in the western region are unable to receive a diagnosis and treatment in a timely manner due to the relatively low economic level and scarcity of resources for medical service systems and facilities. Demographic characteristics, industrial structure, and health habits may also contribute to the high risk of PTB in the western region [9]. Compared with the western region, the central and eastern regions have more developed economies and more advanced medical services [25-27].

The results show that Xinjiang has had the highest PTB notification rate in mainland China for many years [28]. Both the PTB notification case number and notification rate in Xinjiang from 2009 to 2018 gradually increased. A possible reason might be that Xinjiang has adopted a series of measures to screen cases and to control the PTB epidemic. By the end of 2010, Xinjiang had achieved 100% DOTS coverage. In 2013, Xinjiang took the lead in China to implement a new “three-in-one” TB prevention and control service model. This model transformed TB prevention and control activities managed by the local Center for Disease Control (CDC) into an integrated collaboration system involving local CDCs, designated hospitals, and primary medical and health care institutions to coordinate TB prevention and treatment. Policies including centralized isolation treatment for patients during the infectious period and home treatment for patients in the noninfectious period, such as the “centralized medication+nutritious breakfast” approach, have also been implemented in Xinjiang.

The overall PTB notification rate in Tibet showed a decreasing trend, whereas the notification rates in some counties increased. The gradual extension of the TBIMS and Infectious Disease Recording and Reporting Systems (IDRS) in Tibet in recent years is a possible reason for this. As people’s health awareness increases, more people receive health examinations, and many potential PTB cases are detected through health examinations [29]. The majority of Tibet’s population and cities are concentrated in the lower Yarlung Zangbo River Valley in the southeast. Therefore, the RR for PTB gradually increased in a northwest to southeast direction in Tibet. These regions with a lower altitude and a more suitable climate for human living are dense areas of PTB [30]. In addition, the higher RRs for PTB in Xinjiang and Tibet have certain relationships with attention by local governments and people’s living habits [9,30,31]. Many herders have a relatively low educational level, limited economic income, and lack of access to local health services, which promote the continuous spread of PTB and influence PTB prevention and control [29].

The RR for PTB in Qinghai had a relatively stable decreasing trend in the early study period and showed a significant upward trend in 2017 and 2018 [32]. The PTB epidemic in Guizhou has always been at a high level in China, and the main factor affecting PTB transmission is the floating population [33]. The epidemic situation has greatly improved as a result of a shift in the government’s focus in recent years and the strengthening of various TB intervention measures. Therefore, there is a need to strengthen health education, improve the accessibility of health services, and balance the distribution of medical and health resources to improve the rates of TB diagnosis and treatment in the western region.

A high gross domestic product per capita often indicates a high quality of life, a balanced diet, and hygiene knowledge among residents. Tianjin, Beijing, Shanghai, and Shandong were the provinces (municipalities) with the lowest RRs for PTB in mainland China. These provinces have high levels of economic development, high levels of education among the population, high levels of TB knowledge of disease prevention, and high medical standards. In contrast, less developed areas are constrained by economic and cultural factors, and the PTB epidemic is serious in these areas. Considering China’s large population, high proportion of the population with latent TB infection, unbalanced regional development, aging population, increasing floating population, and heavy economic burden, it is necessary to increase the detection, diagnosis, and treatment of TB on the existing basis; optimize the TB prevention and treatment service system; and increase funding to ensure that China achieves the SDGs on schedule by 2030 [34].

There are some limitations that should be considered when interpreting the study findings. First, we did not analyze any type of TB other than PTB, which may have different temporal and spatial patterns of risk. However, considering that PTB cases account for more than 80% of total TB cases, the overall trend should be similar. Second, the specific effects of economic development and medical resources on TB were not considered in the study, which might also vary significantly in different regions.

In conclusion, the overall rate of PTB notification decreased steadily in the last decade in China. However, the notification rates between different provinces and within each province exhibited significant regional inequality. Western China presented a high plateau of the disease burden. These regions also had relatively low declining trends compared to the national overall trend. Improvements in economic and medical service levels in such regions are required to boost PTB case detection and eventually reduce PTB risk in the whole country. These findings improve our understanding of the characteristics of the PTB distribution in China, and provide evidence for the spatial strategy to contain the PTB epidemic.

## Appendix

Multimedia Appendix 1 Classification result based on exp(s_i_) posterior distribution in mainland China from 2009 to 2018. (a) Classification result based on exp(s_i_) posterior distribution at the province level; (b) Classification result based on exp(s_i_) posterior distribution at the county level.

## Abbreviations

BYM: Besag-York-Mollie
CDC: Center for Disease Control
DOTS: directly observed treatment, short-course
ICAR: intrinsic conditional autoregressive
IDRS: Infectious Disease Recording and Reporting Systems
LT: local trend
MCMC: Markov chain Monte Carlo
PTB: pulmonary tuberculosis
RR: relative risk
SDG: sustainable development goal
TB: tuberculosis
TBIMS: National Tuberculosis Information Management System
