# Supplementary figures and images for: Unbalanced Risk of Pulmonary Tuberculosis in China at the Subnational Scale: Spatiotemporal Analysis
Source: JMIR Public Health Surveill. 2022 Jul 1;8(7):e36242. doi: 10.2196/36242 (PMC9288096; doi:10.2196/36242)

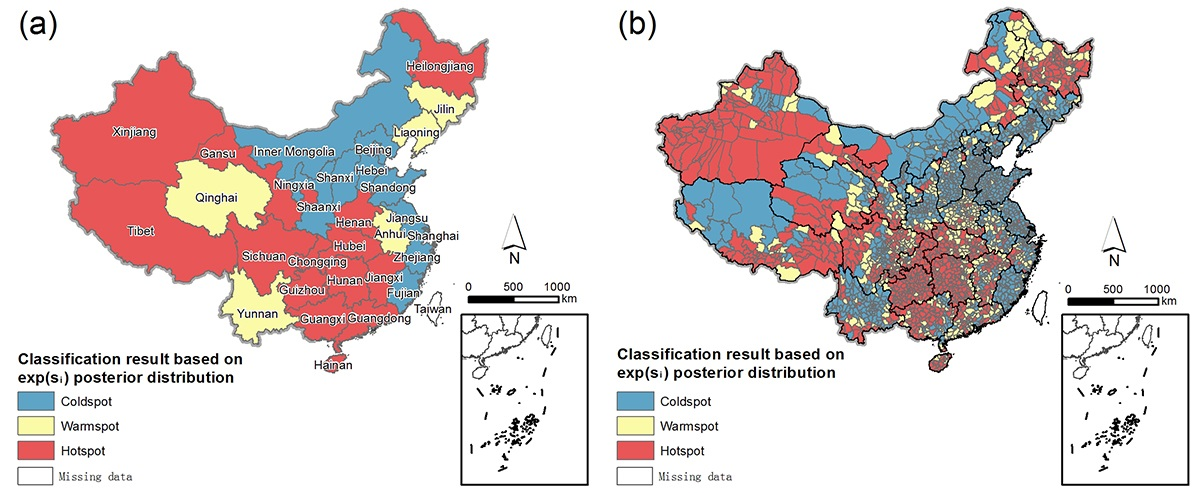

Supplement: Multimedia Appendix 1 [file publichealth_v8i7e36242_app1.png]
